# Supplementary material for: MicroRNA-sensing plasmid system for dynamic control of functional ion channel expression
Source: Mol Ther Nucleic Acids. 2026 Aug 10;37(3):103055. doi: 10.1016/j.omtn.2026.103055 (PMC13524872; doi:10.1016/j.omtn.2026.103055)
Supplement: Supplementary file 1 [file mmc1.pdf]

OMTN, Volume *37*

Supplemental information

MicroRNA-sensing plasmid system for dynamic control of functional ion channel expression

Joschua Geuter, Thomas T. Austin, Jack Corke, Naxi Tian, Jordan Brown, Stephanie Schorge, and Gareth Morris

Table S1
Adjusted P values for individual statistical comparisons from Figure 3F.

**Šídák's multiple comparisons**	**Adjusted P Value**
-80 mV	
Scr vs. Ant-193	>0.9999
Scr vs. 1 nM mim-193	0.6892
Scr vs. 25 nM mim-193	0.9923
Ant-193 vs. 1 nM mim-193	0.6725
Ant-193 vs. 25 nM mim-193	0.9895
1 nM mim-193 vs. 25 nM mim-193	>0.9999
-70 mV	
Scr vs. Ant-193	>0.9999
Scr vs. 1 nM mim-193	0.4482
Scr vs. 25 nM mim-193	0.8325
Ant-193 vs. 1 nM mim-193	0.28
Ant-193 vs. 25 nM mim-193	0.5383
1 nM mim-193 vs. 25 nM mim-193	>0.9999
-60 mV	
Scr vs. Ant-193	>0.9999
Scr vs. 1 nM mim-193	0.1265
Scr vs. 25 nM mim-193	0.496
Ant-193 vs. 1 nM mim-193	0.0256
Ant-193 vs. 25 nM mim-193	0.1327
1 nM mim-193 vs. 25 nM mim-193	>0.9999
-50 mV	
Scr vs. Ant-193	0.9957
**Scr vs. 1 nM mim-193**	**0.0016**
Scr vs. 25 nM mim-193	0.1036
**Ant-193 vs. 1 nM mim-193**	**0.0199**
Ant-193 vs. 25 nM mim-193	0.0634
1 nM mim-193 vs. 25 nM mim-193	>0.9999
-40 mV	
Scr vs. Ant-193	0.5144
**Scr vs. 1 nM mim-193**	**0.0008**
**Scr vs. 25 nM mim-193**	**0.0002**
**Ant-193 vs. 1 nM mim-193**	**0.0026**
**Ant-193 vs. 25 nM mim-193**	**0.0013**
1 nM mim-193 vs. 25 nM mim-193	>0.9999
-30 mV	
Scr vs. Ant-193	0.1605

**Scr vs. 1 nM mim-193**	**0.0005**
**Scr vs. 25 nM mim-193**	**<0.0001**
**Ant-193 vs. 1 nM mim-193**	**<0.0001**
**Ant-193 vs. 25 nM mim-193**	**<0.0001**
1 nM mim-193 vs. 25 nM mim-193	>0.9999

-20 mV

Scr vs. Ant-193	0.0734
**Scr vs. 1 nM mim-193**	**0.0003**
**Scr vs. 25 nM mim-193**	**<0.0001**
**Ant-193 vs. 1 nM mim-193**	**<0.0001**
**Ant-193 vs. 25 nM mim-193**	**<0.0001**
1 nM mim-193 vs. 25 nM mim-193	>0.9999

-10 mV

**Scr vs. Ant-193**	**0.0487**
**Scr vs. 1 nM mim-193**	**0.0002**
**Scr vs. 25 nM mim-193**	**<0.0001**
**Ant-193 vs. 1 nM mim-193**	**<0.0001**
**Ant-193 vs. 25 nM mim-193**	**<0.0001**
1 nM mim-193 vs. 25 nM mim-193	>0.9999

0 mV

**Scr vs. Ant-193**	**0.0326**
**Scr vs. 1 nM mim-193**	**0.0002**
**Scr vs. 25 nM mim-193**	**<0.0001**
**Ant-193 vs. 1 nM mim-193**	**<0.0001**
**Ant-193 vs. 25 nM mim-193**	**<0.0001**
1 nM mim-193 vs. 25 nM mim-193	>0.9999

+10 mV

**Scr vs. Ant-193**	**0.0255**
**Scr vs. 1 nM mim-193**	**0.0002**
**Scr vs. 25 nM mim-193**	**<0.0001**
**Ant-193 vs. 1 nM mim-193**	**<0.0001**
**Ant-193 vs. 25 nM mim-193**	**<0.0001**
1 nM mim-193 vs. 25 nM mim-193	>0.9999

+20 mV

**Scr vs. Ant-193**	**0.0232**
**Scr vs. 1 nM mim-193**	**0.0003**
**Scr vs. 25 nM mim-193**	**<0.0001**
**Ant-193 vs. 1 nM mim-193**	**<0.0001**
**Ant-193 vs. 25 nM mim-193**	**<0.0001**
1 nM mim-193 vs. 25 nM mim-193	>0.9999
