# Supplementary material for: MicroRNA-sensing plasmid system for dynamic control of functional ion channel expression
Source: Mol Ther Nucleic Acids. 2026 Aug 10;37(3):103055. doi: 10.1016/j.omtn.2026.103055 (PMC13524872; doi:10.1016/j.omtn.2026.103055)
Supplement: Supplementary file 2 [file mmc2.pdf]

Molecular Therapy
Nucleic Acids
Brief report

MicroRNA-sensing plasmid system for dynamic control of functional ion channel expression

Joschua Geuter,[1,2] Thomas T. Austin,[3] Jack Corke,[3] Naxi Tian,[1] Jordan Brown,[1] Stephanie Schorge,[1] and Gareth Morris[1,3]

[1]Department of Neuroscience, Physiology and Pharmacology, University College London, Gower Street, London WC1E 6BT, UK; [2]Sainsbury Wellcome Centre, University College London, Howland Street, London W1T 4JG, UK; [3]Division of Neuroscience, University of Manchester, Oxford Road, Manchester M13 9PT, UK

Gene therapy offers the potential for long-term treatment of a range of chronic diseases. However, permanent gene therapy expression may not be desirable. Efforts have been made to create systems that can be switched on/off by stimuli including light, designer drugs, or cellular contexts including increased electrical activity. Here, we designed a plasmid system in which ion channel expression and function are regulated by microRNA (miR)—an endogenous class of short noncoding RNAs which negatively regulate gene expression. We modified an existing voltage-gated potassium channel gene therapy with a binding cassette for miR-193a-3p, and transfected this "miR-193-OFF" system in neuro2A cells. Co-transfection with an inhibitor or mimic of miR-193a-3p, respectively, enhanced or repressed expression of our transgene, assessed using a GFP marker. Using whole-cell voltage clamp, we observed tuneable voltage-gated potassium currents in cells co-transfected with a miR-193a-3p inhibitor/mimic, compared with a non-targeting control oligonucleotide. Together, this demonstrates the concept of a miR-mediated molecular switch which can bias therapeutic ion channel expression based on a specific miR signal. As miRs are a ubiquitous molecular mechanism, our approach could be applied to a wide range of cellular and disease contexts, potentially expanding gene therapy to new patient populations.

INTRODUCTION

Gene therapy offers the prospect of long-lasting therapeutic intervention for patients with otherwise treatment-resistant chronic neurological diseases.[1] In brief, gene therapy typically uses viral vectors to deliver therapeutic sequences into target cells. To reduce off-target effects, in experimental neurology, there is currently an effort to identify ways of selectively targeting the delivery (by injecting directly into the target area, or developing viral serotypes that confer preferential transduction of different cell types), and cell-specific expression using promoters and enhancer elements. These properties offer a level of control over which areas and cell types express gene therapies. These "classical" viral-delivered gene therapy strategies provide long-term constitutive expression of therapeutic transgenes which is broadly suitable for treating chronic neurological diseases. Indeed, gene therapy treatments for rare monogenetic disorders are already clinically available (such as Zolgensma for spinal muscular atrophy[2]), and promising experimental data exist to support the use of gene therapies in various brain conditions, including epilepsy,[1,3,4] Parkinson's disease,[5] and chronic pain.[6]

However, even if restricted to the correct cell types, permanent constitutive gene therapies may still raise important safety concerns: the therapy may not need to be permanently activated, and any adverse effects caused by unnecessary activation would be irreversible in most scenarios (with the possible exception of epilepsies eligible for surgical resection that could physically remove the transduced brain region). There is a critical unmet need for dynamic gene therapy tools that can modify their expression in response to cellular or exogenous stimuli. Pre-clinical experimental approaches include optogenetics,[7] in which light is delivered into the brain to activate light-sensitive ion channels, and chemogenetics,[8,9] in which therapeutic ion channels are activated by a specific ligand which must be administered exogenously. In both of these strategies, the treatment is only active in the presence of an external stimulus, which can be removed if safety concerns arise. However, such approaches do not respond to endogenous changes to cellular environments, and both rely upon constitutive protein expression which may impact the host cell. A recent advance used a promoter sequence from the immediate-early gene *c-Fos* in order to develop an "activity-dependent" gene therapy.[10] In this strategy, enhanced electrical activity in the brain triggers transcription factors which drive expression from the *c-Fos* promoter region, thereby coupling transgene expression to marked increases in brain activity. This has substantial therapeutic potential in diseases such as epilepsy, in which neural hyperexcitability is a hallmark of the condition, and therefore the therapy responds on-demand to the dynamics of the disease itself, albeit

Received 19 January 2026; accepted 6 August 2026;
https://doi.org/10.1016/j.omtn.2026.103055.

Correspondence: Gareth Morris, Division of Neuroscience, University of Manchester, Oxford Road, Manchester M13 9PL, UK.
E-mail: gareth.morris-3@manchester.ac.uk

© 2026 The Author(s). Published by Elsevier Inc. on behalf of The American Society of Gene and Cell Therapy.
This is an open access article under the CC BY license (http://creativecommons.org/licenses/by/4.0/).

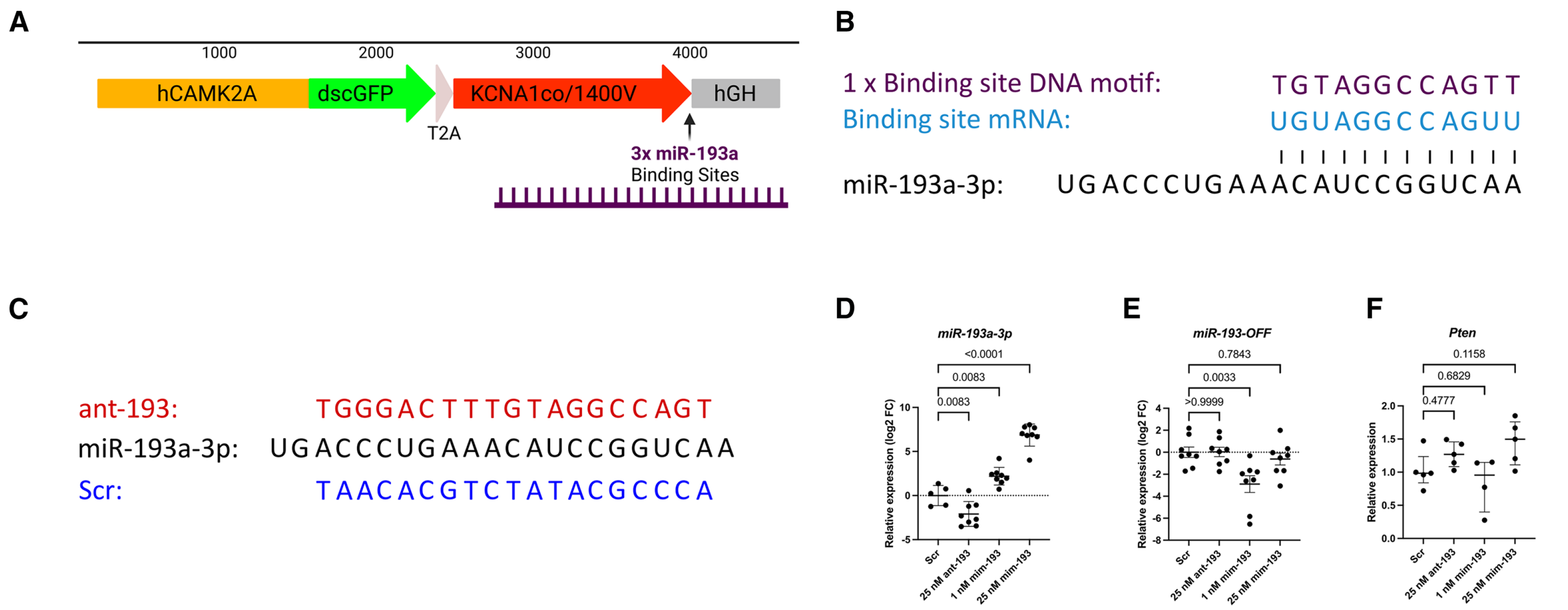

Figure 1. miR-193-OFF plasmid design and miR-193a-3p manipulation

(A) DNA plasmid design for miR-193-OFF. Design is based on a published successful gene therapy[4] and modified to include a synthetic miR-193a-3p binding cassette,[17] based on a naturally occurring motif in the *Pten* gene. (B) Sequences of synthetic binding cassette DNA and RNA, indicating complementarity to miR-193a-3p. (C) Nucleic acid sequences for ant-193, a 19-mer oligonucleotide with full complementarity to miR-193a-3p and non-targeting "Scr" control. (D) RT-qPCR shows ant-193 inhibits miR-193a-3p as expected, and mim-193 boosts expression (left, one-way ANOVA with Holm-Šidák's multiple comparisons). (E) miR-193-OFF transcript level is repressed by 1 nM mim-193, but not changed by ant-193 or 25 nM mim-193 (one-way ANOVA with Holm-Šidák's multiple comparisons). (F) *Pten* transcript levels are not significantly altered by any treatment applied (right, one-way ANOVA with Holm-Šidák's multiple comparisons).

with the caveat that c-Fos is also expressed in healthy neurons, meaning this approach still has risks of interfering with normal c-Fos mediated changes in activity. Novel strategies, responding to other changes in cellular context during brain disease, would represent substantial breakthroughs in the development of disease-driven on-demand gene therapies, especially those where cellular excitability is not a hallmark of disease.

One such cellular context that could be exploited to regulate gene therapy expression is microRNA (miR) expression. miRs are ∼22 nucleotide noncoding RNAs that negatively regulate gene expression through binding complementary sites in the 3′ untranslated region (UTR) of target mRNAs.[11–13] Changing miR levels are closely associated with various brain diseases and in some cases are considered to be biomarkers and/or therapeutic targets.[14–16] Taken together, miRs offer a natural mechanism to modify gene expression, and the expression of miRs themselves is tightly linked to disease contexts. To generate a proof-of-concept, we modified the published "engineered potassium channel" (EKC) epilepsy gene therapy, in which a modified $K_{V1.1}$ channel is used to reduce the activity of excitatory pyramidal neurons and therefore counteract pathological cellular bursting and network hyperactivity.[4] In this work, we cloned a binding cassette for miR-193a-3p[17] into the 3′ UTR of the EKC plasmid. MiR-193a-3p is downregulated in rodent epilepsy models.[15] By incorporating these miR binding sites, we created a system where high miR-193a-3p levels repress ion channel expression through miR-mediated silencing, while reduced miR-193a-3p levels, as in epilepsy models, allow enhanced therapeutic gene expression—effectively creating a miR-responsive molecular switch, which we term "miR-193-OFF." Using a cell-based assay combined with fluorescence imaging and electrophysiological readouts, we demonstrate specific and bidirectional changes in EKC expression and channel function in response to changing miR-193a-3p expression.

RESULTS

miR-193-3p sensitive plasmid design

We based our approach on the previously successful EKC plasmid[4] and inserted a binding cassette for miR-193a-3p into the 3′ UTR, directly following the stop codon (Figures 1A and 1B). The synthetic binding cassette consisted of three consecutive repeats of the sequence TGTAGGCCAGTT, based on a naturally occurring miR-193a-3p binding region in the 3′ UTR of the *Pten* gene.[17] The sequence TGTAGGCCAGTT provides full complementarity, and auxiliary binding, to miR-193-3p′s seed region (Figure 1). Given the natural ability of miRs to repress their target genes, our plasmid (miR-193-OFF) was designed to respond dynamically to changes in miR-193a-3p expression: when miR-193a-3p is low and does not repress the plasmid, the therapy is more active; when miR-193a-3p is high, it represses the plasmid and the therapy is reduced.

Dose-dependent bi-directional changes in plasmid expression with changing miR-193-3p expression

To demonstrate the ability of our plasmid system to respond to changing miR-193a-3p expression, we used cultured neuro2A cells co-transfected with miR and either a locked nucleic acid antimiR (ant-193; inhibiting miR-193a-3p function), a non-targeting scramble control (Scr), or a synthetic miR-193a-3p mimic ("mim-193," overexpressing miR-193a-3p). All nucleic

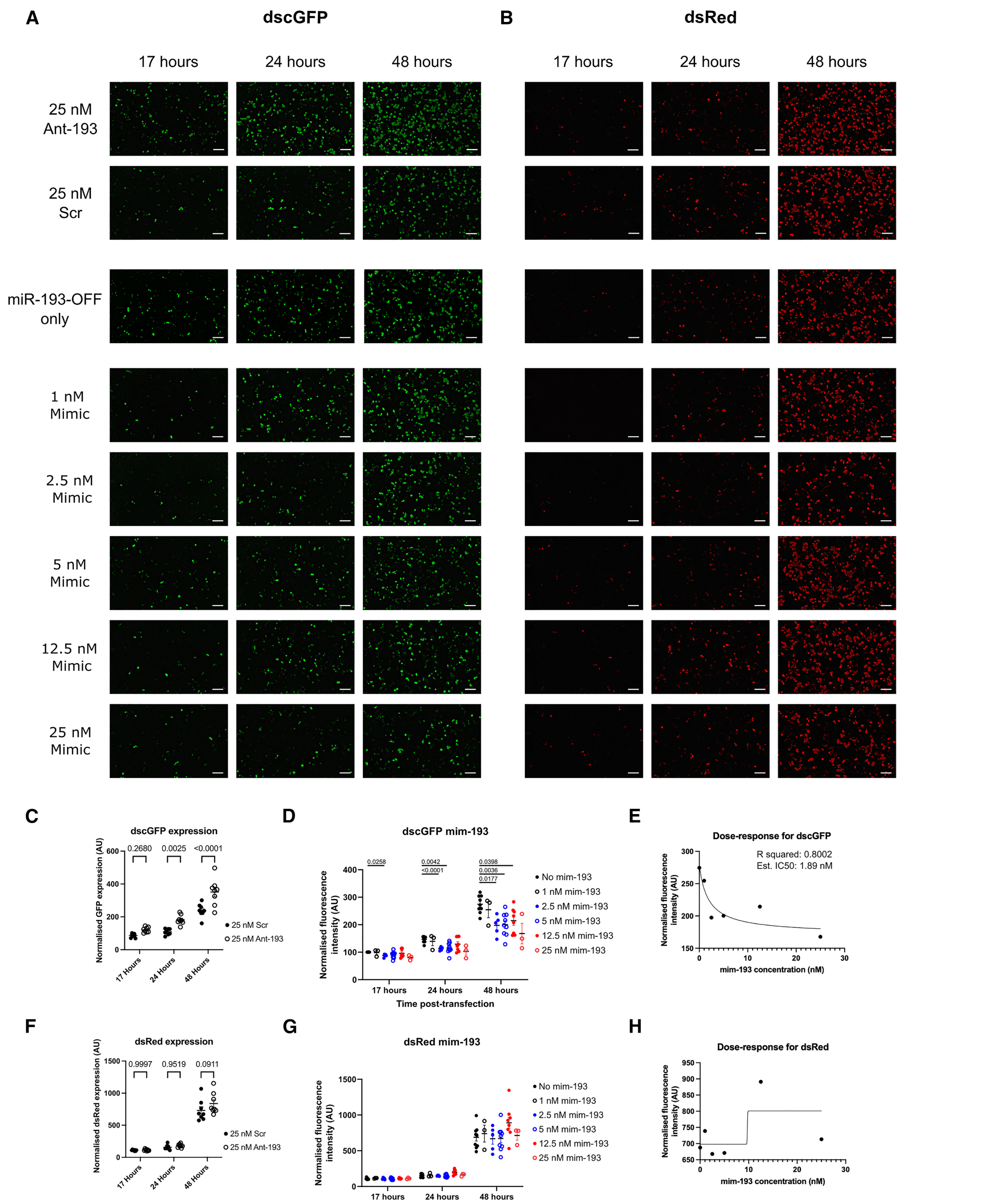

Figure 2. Specific and dose-dependent de-repression or inhibition of miR-193-OFF expression by ant-193 and mim-193

Neuro2a cells were co-transfected with miR-193-OFF (with dscGFP marker), dsRed (non-miR-sensitive control), and 25 nM ant-193a-3p (“ant-193”), 25 nM scrambled non-targeting antimiR control (“Scr”), or 1, 2.5, 5, 12.5, 25 nM mim-193. “miR-193-OFF only” did not include ant-193 or mim-193. (A) Representative live cell fluorescence images

(legend continued on next page)

acid sequences used are presented in Figure 1C. All transfections further contained a miR-193a-3p-insensitive dsRed fluorescent plasmid as a control to demonstrate the specific effects of altered miR-193a-3p on miR-193-OFF. RT-qPCR confirmed presence of endogenous miR-193a-3p in our neuro2A cell line, alongside corresponding inhibition and dose-dependent over-expression when co-transfected with 25 nM ant-193, or 1 nM/25 nM mim-193 (Figure 1D). MicroRNAs can repress gene expression either by target degradation or steric blocking of mRNA translation into protein. Interestingly, our miR-193a-3p manipulation did not lead to direct target transcript degradation (Figure 1E): using the same total RNA as in Figure 1D, we amplified our miR-193-OFF mRNA with specific primers (overlapping the T2A and *Kcna1* elements [Figure 1A]) which cannot amplify any potential native *Kcna1* transcripts. miR-193-OFF values were largely unchanged, with the exception of those treated with 1 nM mim-193, where the predicted decrease in expression was observed. We also measured native *Pten* expression in our cells, given that the miR-193a-3p binding sequence was taken from this gene. We saw no consistent changes in *Pten* transcript levels (Figure 1F). We speculate that miR-193 may have a stronger impact on our synthetic miR-193-OFF which contains a cassette of three miR-193 binding sites, compared with the one binding site on *Pten*.

Since RT-qPCR is not sensitive to mRNA which is blocked but not degraded, we used fluorescence imaging of the destabilized copGFP (dscGFP) reporter marker (Figure 2A) as a semi-quantitative readout of plasmid expression at the protein level. In each experiment, we imaged eight separate random regions of interest for each well at 17, 24, and 48 h post-transfection, measured the fluorescence intensity for each ROI, and averaged these eight values to give an average fluorescence intensity value for each well (with each well as a biological replicate). In control conditions (miR-193-OFF transfected without ant-193, Scr, or mimic; Figure 2A), dscGFP expression increased over time as expected, and miR-193-OFF did not cause toxicity, indicating broad safety. For cells co-transfected with 25 nM ant-193, dscGFP expression was significantly enhanced relative to Scr control within 24 h post-transfection (Figure 2C). The magnitude of differential expression increased at later timepoints. Importantly, dsRed fluorescence was unchanged between ant-193 and Scr treatments (Figures 2B and 2F), highlighting that the changes in gene expression observed were specific to our miR-193a-3p-sensitive plasmid design and not due to general changes in cellular proliferation or protein translation.

To confirm the bi-directional dynamics of our system, we then used varying concentrations of a miR-193a-3p mimic (mim-193), seeking to repress miR-193-OFF expression through mim-193 binding to the plasmid (Figure 2B). Using the same strategy as mentioned earlier, we demonstrated a largely dose-dependent repression of dscGFP signal by mim-193, which emerged at 24 h post-transfection and was more prominent after 48 h (Figures 2B–2E; dose-response R^2 = 0.08002, IC_{50} =∼1.89 nM). Again, miR-193a-3p-insensitive dsRed was not repressed by the mimic (Figures 2G and 2H), showing consistent expression across all concentrations of mim-193 within each time point. Together, these experiments use a semi-quantitative fluorescent protein reporter to show that our miR-193-OFF system mediates bi-directional changes in transgene expression, following corresponding changes in miR-193a-3p levels.

Modifiable voltage-gated potassium channel functional expression in response to miR-193-3p inhibition

Having explored the kinetics of miR-193-OFF using a dscGFP reporter readout, we next verified that we could modify functional ion channel expression using this system, thereby demonstrating that the therapeutic EKC mechanism can be modulated by a miR. Using whole-cell voltage clamp, holding at −60 mV, we first ensured that we could record robust EKC-mediated currents (I_{EKC}) from neuro2A cells transfected with miR-193-OFF (identified by the presence of dscGFP signal), in the absence of miR inhibitors/mimics (Figures 3A–3C). As a control, we investigated whether non-transfected cells (from the same coverslips, but with no observable dscGFP fluorescence) exhibited voltage-sensitive potassium currents. As expected, transfected cells showed significantly higher current density than non-transfected cells (Figure 3B), and only transfected cells showed a non-linear I-V curve (Figure 3C), indicative of voltage-sensitive channel gating. Finally, we assessed EKC current density in cells which were co-transfected with miR-193-OFF and 25 nM ant-193, 1 nM mim-193, 25 nM mim-193, or 25 nM Scr (Figures 3D–3F; Table S1). All groups of cells were able to generate voltage-gated potassium currents (Figure 3D). Cells treated with ant-193 exhibited significantly larger current densities than Scr controls (Figures 3E and 3F; response to 20 mV step: Scr – 165.63 ± 11.63 pA/pF; ant-193 – 256.67 ± 19.47 pA/pF), in line with de-repression of the miR-193-OFF plasmid. Cells treated with both concentrations of mim-193 showed significant repression of voltage-gated potassium current (response to 20 mV step: 1 nM mim-193 = 36.99 ± 16.05 pA/pF; 25 nM mim-193 = 21.24 ± 8.28 pA/pF), indicating reduced translation of miR-193-OFF to protein and in line with

acquired at different timepoints post-transfection show enhanced dscGFP expression in ant-193-treated cells, relative to control, and dose-dependent repression of dscGFP by mim-193. Scale bars, 100 μm. (B) dsRed fluorescence, taken from the same samples and fields of view as the dscGFP images in (A), is not changed by any of the miR-193a-3p manipulations used. (C) Statistical analysis shows that a significant differential expression of dscGFP emerges at 24 h post-transfection and is enhanced at 48 h (RM two-way ANOVA with Šidák's multiple comparisons; *p* values marked on graph). (D) Population fluorescence data (normalized to "no mim-193 17 h" value) for dscGFP demonstrates largely dose-dependent repression of miR-193-OFF by mim-193 (mixed two-way ANOVA with Dunnett's multiple comparisons; significant *p* values at $\alpha = 0.05$ marked on graph). (E) Dose-response analysis using nonlinear regression estimates IC_{50} = 1.89 nM mim-193 with R^2 = 0.8002. (F) Statistical analysis indicates that dsRed, which is not sensitive to miR-193a-3p, does not exhibit significantly different expression at any time point following ant-193 (RM two-way ANOVA with Šidák's multiple comparisons; *p* values marked on graph). (G) dsRed control is insensitive to mim-193 at all timepoints (mixed two-way ANOVA with Dunnett's multiple comparisons; no *p* values were significant at $\alpha = 0.05$). (H) There is no dose-response relationship between mim-193 and dsRed fluorescence.

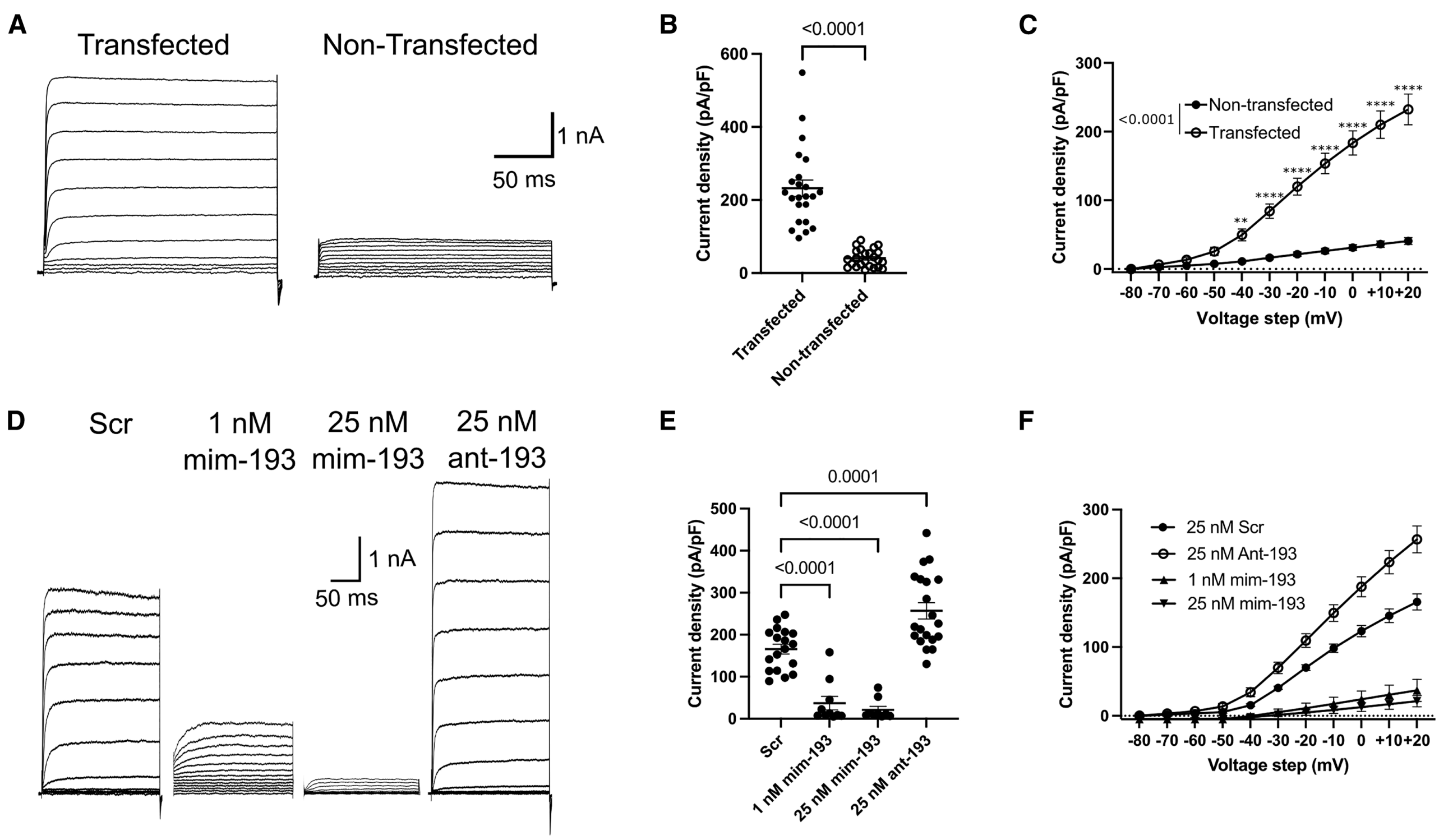

Figure 3. Enhanced voltage-gated potassium channel function in response to miR-193a-3p inhibition

Neuro2A cells were transfected with miR-193-OFF and one of 25 nM ant-193, 1 nM mim-193, 25 nM mim-193, 25 nM Scr, and used for voltage-clamp recordings of V-gated potassium channel currents at 24–48 h post-transfection. (A–C) Cells were transfected with miR-193-OFF only. (A) Only transfected cells (identified using dscGFP label) exhibited voltage-dependent K^+ currents, indicated by representative traces from both transfected and non-transfected cells. (B) Fluorescent cells had significantly greater current density than non-fluorescent cells (scatterplot shows responses to +20 mV voltage step; Student's *t* test). Using the same V-clamp protocol in non-transfected cells, we confirmed that there was no native K_V function in our cell line, shown by a linear I-V relationship (C) Mixed ANOVA with Šidák's multiple comparisons; *p* values marked on graph that reflects ohmic resistance of the cell membrane only. (D–F) in a separate experiment, cells were co-transfected with miR-193-OFF and one of 25 nM ant-193, 1 nM mim-193, 25 nM mim-193, 25 nM Scr. In each group, we selected transfected (dscGFP-positive) cells for recordings. (D) Representative voltage-gated K^+ currents in cells from each group. Ant-193-treated cells showed markedly enhanced I_{Kv} relative to Scr, whereas both mim-193 groups had reduced current density. (E) Responses to +20 mV voltage step (one-way ANOVA with Dunnett's multiple comparisons); (F) I-V curve for all voltage steps (RM-two way ANOVA with Šidák's, *p* values for multiple comparisons in Table S1). Together, these experiments demonstrate that ion channel function in our plasmid is modulated by changing miR-193a-3p expression.

dscGFP reporter data in Figure 2. Together, these data indicate that functional EKC expression and currents can be controlled bi-directionally via miR levels. This finding shows that we can create dynamic synthetic gene delivery systems by incorporating miR binding cassettes—designed to respond to particular cellular contexts—into the 3′ UTR.

DISCUSSION

Here, we demonstrate a dynamic gene therapy expression system where synthetic ion channel expression can be modulated bi-directionally in response to endogenous miR signals. As a proof-of-concept, we modified an existing promising pre-clinical *Kcna1*-based epilepsy gene therapy plasmid, adding a new level of control mediated by miR-193a-3p.

A new level of gene therapy control

Concerns remain that constitutive "classical" gene therapy expression may pose safety risks since adverse effects could not be reversed in the majority of cases. As such, various gene therapy strategies have been developed which respond to certain stimuli, offering a level of control over their expression. Strategies include optogenetics and chemogenetics and, more recently, cell autonomous approaches such as "activity-dependent" gene therapy.[10] The latter circumvents the need for exogenous control of the gene therapy by using an activity-driven promoter to bias gene therapy expression to the most active neurons—a strategy particularly amenable to epilepsy. In this work, we developed an expression system in which a gene therapy can be modulated by changing levels of an endogenous miR. This has several advantages. First, it does not require exogenous activation (unlike opto- and chemo-genetics), removing the need for invasive delivery of light to potentially deep brain structures (optogenetics), or administration of a designer ligand (chemogenetics). Second, modifiable transcript expression circumvents the constitutive protein expression which is required for opto/chemogenetics and which could be harmful to cells. Third, miR-regulation of gene expression is ubiquitous to all cells and, therefore, our strategy may have utility in

a broad range of tissues, compared with an activity-dependent strategy which exploits enhanced activity in excitable cells only. Fourth, there are many hundreds of different miRs in mouse and thousands in human. Thus, our initial proof-of-concept could be reconfigured to respond to changes in specific individual miRs or combinations of them. Strategic selection of a miR biomarker signature may be highly context- or disease-specific.

A novel system to modulate ion channel expression

Certain miRs are known to have physiological roles in fine-tuning the expression of genes encoding for ion channels.[18] For example, miR-324-5p represses *Kcnd2,*[19] encoding K_V4.2, and miR-335-5p targets multiple voltage-gated sodium channel genes.[20] Therefore, it is known that miRs form part of a natural homeostatic mechanism to maintain neuronal excitability within a physiological range.[18,21] Here, we exploit this natural function of miR to add a layer of control to the expression of an exogenous therapeutic ion channel plasmid. Previous studies have used a similar approach as a semi-quantitative readout of miR expression by cloning combinations of miR binding sites into the 3′ UTR of luciferase reporter gene.[22,23] This strategy permitted the authors to study the temporal kinetics of specific miRs *in vivo*. However, to our knowledge, our study is the first to demonstrate a dynamic exogenous ion channel expression system using a miR-based switching mechanism.

Design considerations for a miR-based tuning system

Our approach is reconfigurable with different promoters, transgenes, and miR-binding cassettes and is therefore amenable to a wide range of applications where expression of a particular miR is a proxy of a cellular state in which expression is desirable. An important consideration is cell-type specificity. When selecting a miR to control the tuning mechanism, it is critical to ensure that the chosen miR is expressed in the targeted cells. This can be matched to cell-specific promoter systems for enhanced selectivity. In this case, we have chosen miR-193 because we have previously shown this miR to be upregulated in several models of epilepsy,[15] making it a strong candidate for driving selective expression in response to seizures. As miRs are dynamically regulated in many disease states, this approach means that miR-OFF may be translatable to additional clinical needs.

Notably, our system appeared to favor miR-193-OFF repression via a steric blocking mechanism, rather than transcript degradation—indicated by changes at the protein level (fluorescent reporter and patch clamp) but not RNA transcript expression (RT-qPCR). This may be preferable because an intact therapeutic transcript is available upon miR-193 inhibition, rather than the cell having to transcribe a new mRNA copy. For applications where mRNA degradation is preferable, miR binding sequences may have to be optimized to favor this mechanism.[13]

Limitations

Whilst our system is able to mediate dynamic transgene gene expression, the system was not fully switched off even in the presence of relatively high mim-193 concentrations (Figures 2A and 2C). This is consistent with the physiological roles of miRs, which fine-tune gene expression[14,24] rather than acting as binary ON/OFF switches. However, in certain applications, it may be preferable to fully switch off transgene expression when it is not required. This is beyond the functionality of our proof-of-concept, but it is conceivable that miR-mediated transgene repression could be enhanced by different configurations of binding sites.

Although not intended as a primary mechanism of our system, miR-193-OFF may lead to "sponging" of the miR which is used to trigger the switching mechanism. Briefly, miRs may form stable duplexes with their mRNA targets, therefore "sponging" the miR and preventing it from engaging with its physiological targets. As this approach develops toward the clinic, wider bioinformatic and transcriptomic/proteomic analyses will be required to assess any broader effects on gene expression. However, given the physiological role of miRs, we would anticipate any off-target effects to be subtle.

Finally, our proof-of-concept used a mouse neuroblastoma cell line. Further work to develop this concept toward potential clinical translation will require testing in human cell lines as well as using *in vivo* models. Testing in surgically resected human epileptic tissues may offer an alternative route for validation *in vitro*.[25]

Conclusions

We have demonstrated proof-of-concept for a dynamic and bidirectional miR-modulated transgene expression system for ion channels. This raises the prospect of gene therapy expression which is highly specific to certain cellular or disease contexts, alleviating potential safety concerns about the permanent constitutive expression of gene therapy plasmids.

MATERIALS AND METHODS

miR-193-OFF plasmid development

We based our approach on the EKC plasmid, developed in Stephanie Schorge's lab,[4] which expresses a modified and codon-optimized version of the voltage-gated potassium channel K_V1.1. In this work, EKC was expressed within an AAV2 plasmid backbone, containing inverted terminal repeats (ITRs), and under the control of a human CAMK2A promoter. For optical readout of transgene expression, destabilized dscGFP was coupled to EKC via a T2A peptide.

We identified the miR-193a-3p binding motif GGCCAGU from the 3′ UTR of the human *PTEN* gene,[17] which binds the miR-193a-3p seed region 2-ACUGGCC-8. We added the auxiliary binding nucleotides to create a full 1× miR-193 binding motif: TGTAG GCCAGTT, and repeated this 3× to form a miR-193 binding cassette: TGTAGGCCAGTTTGTAGGCCAGTTTGTAGGCCA GTT. This cassette was cloned into the EKC plasmid immediately downstream of the ATG stop codon, to place the expression of the exogenous EKC plasmid under the influence of miR-193a-3p ("miR-193-OFF"). The full miR-193-OFF EKC plasmid is shown diagrammatically in Figure 1.

Cell culture and transfection

Mouse neuro2a (N2A) cells were cultured in a humidified 5% CO_2 atmosphere at 37°C in DMEM (Gibco 41966-029) supplemented with 10% fetal bovine serum (FBS; Sigma F7524), 1% non-essential amino acids (Sigma M7145-100 mL), and 1% penicillin/streptomycin (Gibco 15140-122). Cells were split every 2–3 days to maintain logarithmic growth and kept for up to 20 passages. For splitting, cells were washed with 2 mL Hank's balanced salt solution (HBSS; Gibco 14170-112) and incubated for 5 min at 37°C with 0.05% trypsin-EDTA (Gibco 25300-054). Trypsin reaction was halted using 3 mL prewarmed DMEM + FBS. After centrifugation (1 min, 1,000 rpm), cells were resuspended in 1 mL cell culture medium. Subsequently, one-eighth of the cell solution was re-seeded in a fresh T75 flask containing 20 mL medium.

Cells for transfection were cultured as described and seeded in a 6-well dish with 4 mL of cell culture medium 24 h before transfection, sufficient to achieve ∼30% total confluency during transfection, which facilitated optimal transfection efficiency for this work. Chemical transfections used 6 μL TurboFect transfection reagent (ThermoFisher, R0534) in 400 μL serum-free OptiMEM (Gibco 31985-062) per well. Plasmid DNA for miR-193-OFF and dsRed (as miR-insensitive control) were added to achieve a final concentration of 0.5 μg DNA per plasmid per well. Locked nucleic acid miR inhibitors with phosphorothioate backbone were purchased from Qiagen with the following sequences: antimiR-193a-3p ("Ant-193"; 5′-TGGGACTTTGTAGGCCAGT-3′) and non-targeting scrambled control ("Scr"; 5′-TAACACGTCTATACGCCCA-3′). Experiments involving miR mimic used synthetic miR-193a-3p ("mim-193") with the sequence 5′-AACUGGCCUACAAAGUCCCAGU-3′.

The respective oligonucleotides were added to the transfection solution to achieve a final concentration in the well of 12.5 or 25 nM for ant-193 or Scr, or 1, 2.5, 5, 12.5, or 25 nM for mim-193, based on the manufacturer's guidelines. After a 20-min incubation, the transfection solutions were added to the wells. Transfection medium was replaced the following morning, ∼17 h post-transfection, prior to initial imaging. All transfections were conducted blinded to the experimental conditions.

RNA extraction

Culture medium was aspirated from each well, and cells were washed once with phosphate-buffered saline (PBS). RNA extraction was performed using Trizol and following standard protocols (e.g., Venø et al.[15]). RNA concentration and purity were determined using a NanoDrop spectrophotometer, and samples were stored at −70°C until further use. To remove contaminating genomic DNA, RNA samples were treated using Thermo Scientific DNase I, RNase-free (cat. no. EN0521).

Reverse transcription and quantitative real-time PCR

For mRNA analysis, cDNA was synthesized using the Thermo Scientific RevertAid First Strand cDNA Synthesis Kit (cat. no. K1621/K1622), with random hexamer primers according to the manufacturer's instructions. For miR analysis, cDNA was generated using the Applied Biosystems TaqMan Advanced miR cDNA Synthesis Kit (cat. no. A28007), according to the manufacturer's protocol, using 10 ng RNA per sample.

mRNA expression was quantified by SYBR Green real-time PCR using gene-specific primers on a LightCycler 480 II Real-Time PCR System (Roche). Relative mRNA expression was calculated using the $2^{-\Delta\Delta Ct}$ method following normalization to *Gapdh*. Primer sequences: *miR-193-OFF*: F – 5′-GGAGAATCCCGGCCCTATGA-3′, R – 5′-GCC TGTCTGGGATAGCTGC-3′ (overlaps the kcna1 and T2A linker elements); *Pten:* F – 5′-GACCATAACCCACCACAGC-3′, R – 5′-CAT TACACCAGTCCGTCCCT-3′; *Gapdh:* F – 5′- AACTTTGGCATTGT GGAAGG-3′, R – 5′-ACACATTGGGGGTAGGAAC-3′. MicroRNA expression was quantified using TaqMan Advanced miR Assays (Applied Biosystems) together with TaqMan Fast Advanced Master Mix. Relative miR expression was calculated following normalization to the endogenous reference miR *miR-26a-5p*.

Fluorescence imaging

Transfected cells were imaged at 17-, 24-, and 48-h post-transfection using an Olympus IX71 microscope (10× objective) equipped with a Kiralux Camera and ThorCam software (version 3.7.0.6). Eight individual regions of interest were imaged for each condition and time point, and values presented are an average of the eight images. Imaging sites within the well were randomly selected and ensured to contain comparable cell density across conditions. Sequential dsRed (545/583 nm excitation/emission) and dscGFP (470/508 nm excitation/emission) fluorescence images were obtained using the CoolLED fluorescence illuminator. Image acquisition was conducted blinded to the experimental conditions within each well.

Image analysis

Fluorescence intensity in dscGFP-positive cells was quantified using FIJI[26] (version 1.54) by calculating integrated density, which summed pixel intensities across the image and divided by image area to present total fluorescence. Background fluorescence was subtracted by creating a Gaussian-blurred version of the fluorescence image (sigma width 30 pixels) and subtracting it from the original image prior to integrated density calculation. dsRed images were analyzed using integrated density calculation, with background fluorescence derived from the minimum fluorescent intensity within each image and subtracted, using skimage (version 0.21.0) and NumPy (version 1.25.1) packages in Python (version 3.9). Mean integrated density was calculated across eight replicates for each condition and time point. To normalize between transfections, mean integrated density values were divided by the respective 17-h negative control value, presenting all data as a percentage of control minimal fluorescence for each trial.

Patch clamp

Neuro2A cells were seeded onto 9 mm borosilicate glass coverslips and transfected with miR-193-OFF, either alone or accompanied

by ant-193, mim-193 or Scr, following the procedures outlined as mentioned earlier. Coverslips were placed in a Nikon Eclipse Ti-U inverted microscope chamber equipped with Plan Fluor 10× Ph1DL and S Plan Fluor ELWD 40× DIC N1 objectives (Nikon). Coverslips were immersed in a static extracellular solution comprising (in mM): 140 NaCl, 4 KCl, 1.8 $CaCl_2$, 2 $MgCl_2$, and 10 HEPES, pH adjusted to 7.34–7.37 with NaOH, osmolality 293 mmol/kg.

~5 MΩ borosilicate glass micropipettes were filled with an intracellular solution consisting of (in mM): 140 KCl, 1 $MgCl_2$, 0.5 $CaCl_2$, 10 HEPES, pH adjusted to 7.3 with KOH, and osmolality 309 mmol/kg. V-gated potassium channel currents were recorded using whole-cell voltage-clamp configuration, monitoring cell capacitance and series resistance. Recordings with series resistance >20 MΩ were discarded. Series resistance compensation was not applied. Cells were held at a resting potential of −80 mV for 50 ms, followed by a 200 ms voltage step delivered in 10 mV increments up to +20 mV. Each current step was followed by a 40 ms hyperpolarizing step to −100 mV before returning to baseline for an additional 40 ms. Signals were amplified using an Axopatch 200B amplifier (Molecular Devices) and Axopatch 203BU headstage, lowpass filtered at 2 kHz, and sampled at 10 kHz using Clampex Software (version 10.7) and a Digidata 1440A (Molecular Devices). Prior to data acquisition, cells were allowed to equilibrate for at least three minutes. Recordings were conducted at room temperature.

Statistical analysis

All statistical analyses were conducted using either Python (version 3.9) with the following packages and versions: pyABF (2.3.7), NumPy (1.25.1), pandas (2.0.3), stasmodels (0.14.0), Seaborn (0.12.2), Matplotlib (3.7.2), SciPy (1.11.1), skimage (0.21.0) (code for these analyses at https://github.com/Joschua21/miRsense_OFF.git), or GraphPad Prism (v10).

Data were tested for normality using Shapiro-Wilk test, and parametric or non-parametric tests were selected accordingly. Singular outliers were identified using a Grubbs test. Individual statistical tests used are listed in the figure legends for each *p* value presented. Data are presented as mean ± SEM.

DATA AND CODE AVAILABILITY

Raw data are available upon reasonable written request to the corresponding author.

ACKNOWLEDGMENTS

G.M. and T.T.A. were funded by the Epilepsy Research Institute (F2102 Morris, 2501P Morris). G.M. was funded by the Royal Society (RGS\R2\222326). S.S. and N.T. were funded by the UKRI Medical Research Council (MR/V034758/1). J.C. was funded by an Epilepsy Research Institute Doctoral Training Center (DTC2406 North West).

AUTHOR CONTRIBUTIONS

J.G., T.T.A., J.C., N.T., and J.B. performed experiments and analyzed data. S.S. and G.M. designed the study. J.G. and G.M. wrote the manuscript. All authors approved the final version of the manuscript.

DECLARATION OF INTERESTS

S.S. holds a patent on the original EKC construct.

SUPPLEMENTAL INFORMATION

Supplemental information can be found online at https://doi.org/10.1016/j.omtn.2026.103055.

REFERENCES

1. Morris, G., and Schorge, S. (2022). Gene Therapy for Neurological Disease: State of the Art and Opportunities for Next-generation Approaches. Neuroscience *490*, 309–314. https://doi.org/10.1016/j.neuroscience.2022.03.010.
2. Day, J.W., Finkel, R.S., Chiriboga, C.A., Connolly, A.M., Crawford, T.O., Darras, B.T., Iannaccone, S.T., Kuntz, N.L., Peña, L.D.M., Shieh, P.B., et al. (2021). Onasemnogene abeparvovec gene therapy for symptomatic infantile-onset spinal muscular atrophy in patients with two copies of SMN2 (STR1VE): an open-label, single-arm, multicentre, phase 3 trial. Lancet Neurol. *20*, 284–293. https://doi.org/10.1016/S1474-4422(21)00001-6.
3. Kullmann, D.M., Schorge, S., Walker, M.C., and Wykes, R.C. (2014). Gene therapy in epilepsy—is it time for clinical trials? Nat. Rev. Neurol. *10*, 300–304. https://doi.org/10.1038/nrneurol.2014.43.
4. Snowball, A., Chabrol, E., Wykes, R.C., Shekh-Ahmad, T., Cornford, J.H., Lieb, A., Hughes, M.P., Massaro, G., Rahim, A.A., Hashemi, K.S., et al. (2019). Epilepsy gene therapy using an engineered potassium channel. J. Neurosci. *39*, 3159–3169. https://doi.org/10.1523/JNEUROSCI.1143-18.2019.
5. Palfi, S., Gurruchaga, J.M., Lepetit, H., Howard, K., Ralph, G.S., Mason, S., Gouello, G., Domenech, P., Buttery, P.C., Hantraye, P., et al. (2018). Long-Term Follow-Up of a Phase I/II Study of ProSavin, a Lentiviral Vector Gene Therapy for Parkinson's Disease. Hum. Gene Ther. Clin. Dev. *29*, 148–155. https://doi.org/10.1089/humc.2018.081.
6. Snowball, A., and Schorge, S. (2015). Changing channels in pain and epilepsy: Exploiting ion channel gene therapy for disorders of neuronal hyperexcitability. FEBS Lett. *589*, 1620–1634. https://doi.org/10.1016/j.febslet.2015.05.004.
7. Deisseroth, K., Feng, G., Majewska, A.K., Miesenböck, G., Ting, A., and Schnitzer, M.J. (2006). Next-generation optical technologies for illuminating genetically targeted brain circuits. J. Neurosci. *26*, 10380–10386. https://doi.org/10.1523/JNEUROSCI.3863-06.2006.
8. Armbruster, B.N., Li, X., Pausch, M.H., Herlitze, S., and Roth, B.L. (2007). Evolving the lock to fit the key to create a family of G protein-coupled receptors potently activated by an inert ligand. Proc. Natl. Acad. Sci. USA *104*, 5163–5168. https://doi.org/10.1073/pnas.0700293104.
9. Weston, M., Kaserer, T., Wu, A., Mouravlev, A., Carpenter, J.C., Snowball, A., Knauss, S., Von Schimmelmann, M., During, M.J., Lignani, G., et al. (2019). Olanzapine: A potent agonist at the hM4D(Gi) DREADD amenable to clinical translation of chemogenetics. Sci. Adv. *5*, eaaw1567–eaaw1569. https://doi.org/10.1126/sciadv.aaw1567.
10. Qiu, Y., O'Neill, N., Maffei, B., Zourray, C., Almacellas-Barbanoj, A., Carpenter, J.C., Jones, S.P., Leite, M., Turner, T.J., Moreira, F.C., et al. (2022). On-demand cell-autonomous gene therapy for brain circuit disorders. Science *378*, 523–532. https://doi.org/10.1126/science.abq6656.
11. Bartel, D.P. (2018). Metazoan MicroRNAs. Cell *173*, 20–51. https://doi.org/10.1016/j.cell.2018.03.006.
12. Bartel, D.P. (2004). MicroRNAs: Genomics, Biogenesis, Mechanism, and Function. Cell *116*, 281–297. https://doi.org/10.1016/S0092-8674(04)00045-5.
13. Bartel, D.P. (2009). MicroRNAs: Target Recognition and Regulatory Functions. Cell *136*, 215–233. https://doi.org/10.1016/j.cell.2009.01.002.
14. Morris, G., O'Brien, D., and Henshall, D.C. (2021). Opportunities and challenges for microRNA-targeting therapeutics for epilepsy. Trends Pharmacol. Sci. *42*, 605–616. https://doi.org/10.1016/j.tips.2021.04.007.
15. Venø, M.T., Reschke, C.R., Morris, G., Connolly, N.M.C., Su, J., Yan, Y., Engel, T., Jimenez-Mateos, E.M., Harder, L.M., Pultz, D., et al. (2020). A systems approach delivers a functional microRNA catalog and expanded targets for seizure suppression

in temporal lobe epilepsy. Proc. Natl. Acad. Sci. USA *117*, 15977–15988. https://doi.org/10.1073/pnas.1919313117.

16. Brennan, G.P., Bauer, S., Engel, T., Jimenez-Mateos, E.M., Del Gallo, F., Hill, T.D.M., Connolly, N.M.C., Costard, L.S., Neubert, V., Salvetti, B., et al. (2020). Genome-wide microRNA profiling of plasma from three different animal models identifies biomarkers of temporal lobe epilepsy. Neurobiol. Dis. *144*, 105048. https://doi.org/10.1016/j.nbd.2020.105048.
17. Jian, B., Li, Z., Xiao, D., He, G., Bai, L., and Yang, Q. (2016). Downregulation of microRNA-193-3p inhibits tumor proliferation migration and chemoresistance in human gastric cancer by regulating PTEN gene. Tumor Biol. *37*, 8941–8949. https://doi.org/10.1007/s13277-015-4727-x.
18. Morris, G. (2023). MicroRNAs – small RNAs with a big influence on brain excitability. J. Physiol. *601*, 1711–1718. https://doi.org/10.1113/JP283719.
19. Gross, C., Yao, X., Engel, T., Tiwari, D., Xing, L., Rowley, S., Danielson, S.W., Thomas, K.T., Jimenez-Mateos, E.M., Schroeder, L.M., et al. (2016). MicroRNA-Mediated Downregulation of the Potassium Channel Kv4.2 Contributes to Seizure Onset. Cell Rep. *17*, 37–45. https://doi.org/10.1016/j.celrep.2016.08.074.
20. Heiland, M., Connolly, N.M.C., Mamad, O., Nguyen, N.T., Kesavan, J.C., Langa, E., Fanning, K., Sanfeliu, A., Yan, Y., Su, J., et al. (2023). MicroRNA-335-5p suppresses voltage-gated sodium channel expression and may be a target for seizure control. Proc. Natl. Acad. Sci. *120*, e2216658120. https://doi.org/10.1073/pnas.2216658120.
21. Gross, C., and Tiwari, D. (2018). Regulation of Ion Channels by MicroRNAs and the Implication for Epilepsy. Curr. Neurol. Neurosci. Rep. *18*, 60. https://doi.org/10.1007/s11910-018-0870-2.
22. Ezzine, S., Vassaux, G., Pitard, B., Barteau, B., Malinge, J.M., Midoux, P., Pichon, C., and Baril, P. (2013). RILES, a novel method for temporal analysis of the in vivo regulation of miRNA expression. Nucleic Acids Res. *41*, e192. https://doi.org/10.1093/nar/gkt797.
23. Simion, V., Henriet, E., Juric, V., Aquino, R., Loussouarn, C., Laurent, Y., Martin, F., Midoux, P., Garcion, E., Pichon, C., and Baril, P. (2020). Intracellular trafficking and functional monitoring of miRNA delivery in glioblastoma using lipopolyplexes and the miRNA-ON RILES reporter system. J. Control. Release *327*, 429–443. https://doi.org/10.1016/j.jconrel.2020.08.028.
24. Hobert, O. (2007). miRNAs Play a Tune. Cell *131*, 22–24. https://doi.org/10.1016/j.cell.2007.09.031.
25. Morris, G., Rowell, R., and Cunningham, M. (2021). Limitations of animal epilepsy research models: Can epileptic human tissue provide translational benefit? ALTEX *38*, 451–462. https://doi.org/10.14573/altex.2007082.
26. Schindelin, J., Arganda-Carreras, I., Frise, E., Kaynig, V., Longair, M., Pietzsch, T., Preibisch, S., Rueden, C., Saalfeld, S., Schmid, B., et al. (2012). Fiji: an open-source platform for biological-image analysis. Nat. Methods *9*, 676–682. https://doi.org/10.1038/nmeth.2019.

OMTN, Volume *37*

Supplemental information

MicroRNA-sensing plasmid system for dynamic control of functional ion channel expression

Joschua Geuter, Thomas T. Austin, Jack Corke, Naxi Tian, Jordan Brown, Stephanie Schorge, and Gareth Morris

Table S1
Adjusted P values for individual statistical comparisons from Figure 3F.

**Šídák's multiple comparisons**	**Adjusted P Value**
-80 mV	
Scr vs. Ant-193	>0.9999
Scr vs. 1 nM mim-193	0.6892
Scr vs. 25 nM mim-193	0.9923
Ant-193 vs. 1 nM mim-193	0.6725
Ant-193 vs. 25 nM mim-193	0.9895
1 nM mim-193 vs. 25 nM mim-193	>0.9999
-70 mV	
Scr vs. Ant-193	>0.9999
Scr vs. 1 nM mim-193	0.4482
Scr vs. 25 nM mim-193	0.8325
Ant-193 vs. 1 nM mim-193	0.28
Ant-193 vs. 25 nM mim-193	0.5383
1 nM mim-193 vs. 25 nM mim-193	>0.9999
-60 mV	
Scr vs. Ant-193	>0.9999
Scr vs. 1 nM mim-193	0.1265
Scr vs. 25 nM mim-193	0.496
Ant-193 vs. 1 nM mim-193	0.0256
Ant-193 vs. 25 nM mim-193	0.1327
1 nM mim-193 vs. 25 nM mim-193	>0.9999
-50 mV	
Scr vs. Ant-193	0.9957
**Scr vs. 1 nM mim-193**	**0.0016**
Scr vs. 25 nM mim-193	0.1036
**Ant-193 vs. 1 nM mim-193**	**0.0199**
Ant-193 vs. 25 nM mim-193	0.0634
1 nM mim-193 vs. 25 nM mim-193	>0.9999
-40 mV	
Scr vs. Ant-193	0.5144
**Scr vs. 1 nM mim-193**	**0.0008**
**Scr vs. 25 nM mim-193**	**0.0002**
**Ant-193 vs. 1 nM mim-193**	**0.0026**
**Ant-193 vs. 25 nM mim-193**	**0.0013**
1 nM mim-193 vs. 25 nM mim-193	>0.9999
-30 mV	
Scr vs. Ant-193	0.1605

**Scr vs. 1 nM mim-193**	**0.0005**
**Scr vs. 25 nM mim-193**	**<0.0001**
**Ant-193 vs. 1 nM mim-193**	**<0.0001**
**Ant-193 vs. 25 nM mim-193**	**<0.0001**
1 nM mim-193 vs. 25 nM mim-193	>0.9999
-20 mV	
Scr vs. Ant-193	0.0734
**Scr vs. 1 nM mim-193**	**0.0003**
**Scr vs. 25 nM mim-193**	**<0.0001**
**Ant-193 vs. 1 nM mim-193**	**<0.0001**
**Ant-193 vs. 25 nM mim-193**	**<0.0001**
1 nM mim-193 vs. 25 nM mim-193	>0.9999
-10 mV	
**Scr vs. Ant-193**	**0.0487**
**Scr vs. 1 nM mim-193**	**0.0002**
**Scr vs. 25 nM mim-193**	**<0.0001**
**Ant-193 vs. 1 nM mim-193**	**<0.0001**
**Ant-193 vs. 25 nM mim-193**	**<0.0001**
1 nM mim-193 vs. 25 nM mim-193	>0.9999
0 mV	
**Scr vs. Ant-193**	**0.0326**
**Scr vs. 1 nM mim-193**	**0.0002**
**Scr vs. 25 nM mim-193**	**<0.0001**
**Ant-193 vs. 1 nM mim-193**	**<0.0001**
**Ant-193 vs. 25 nM mim-193**	**<0.0001**
1 nM mim-193 vs. 25 nM mim-193	>0.9999
+10 mV	
**Scr vs. Ant-193**	**0.0255**
**Scr vs. 1 nM mim-193**	**0.0002**
**Scr vs. 25 nM mim-193**	**<0.0001**
**Ant-193 vs. 1 nM mim-193**	**<0.0001**
**Ant-193 vs. 25 nM mim-193**	**<0.0001**
1 nM mim-193 vs. 25 nM mim-193	>0.9999
+20 mV	
**Scr vs. Ant-193**	**0.0232**
**Scr vs. 1 nM mim-193**	**0.0003**
**Scr vs. 25 nM mim-193**	**<0.0001**
**Ant-193 vs. 1 nM mim-193**	**<0.0001**
**Ant-193 vs. 25 nM mim-193**	**<0.0001**
1 nM mim-193 vs. 25 nM mim-193	>0.9999
